# Narrow band perfect absorber for maximum localized magnetic and electric field enhancement and sensing applications

**DOI:** 10.1038/srep24063

**Published:** 2016-04-05

**Authors:** Zhengdong Yong, Senlin Zhang, Chensheng Gong, Sailing He

**Affiliations:** 1 Centre for Optical and Electromagnetic Research, State Key Laboratory of Modern Optical Instrumentations, Zhejiang University, Hangzhou 310058, China; 2 Department of Electromagnetic Engineering, School of Electrical Engineering, Royal Institute of Technology (KTH), S-100 44 Stockholm, Sweden

## Abstract

Plasmonics offer an exciting way to mediate the interaction between light and matter, allowing strong field enhancement and confinement, large absorption and scattering at resonance. However, simultaneous realization of ultra-narrow band perfect absorption and electromagnetic field enhancement is challenging due to the intrinsic high optical losses and radiative damping in metals. Here, we propose an all-metal plasmonic absorber with an absorption bandwidth less than 8 nm and polarization insensitive absorptivity exceeding 99%. Unlike traditional Metal-Dielectric-Metal configurations, we demonstrate that the narrowband perfect absorption and field enhancement are ascribed to the vertical gap plasmonic mode in the deep subwavelength scale, which has a high quality factor of 120 and mode volume of about 10^−4^ × (*λ*_*res*_/*n*)^3^. Based on the coupled mode theory, we verify that the diluted field enhancement is proportional to the absorption, and thus perfect absorption is critical to maximum field enhancement. In addition, the proposed perfect absorber can be operated as a refractive index sensor with a sensitivity of 885 nm/RIU and figure of merit as high as 110. It provides a new design strategy for narrow band perfect absorption and local field enhancement, and has potential applications in biosensors, filters and nonlinear optics.

Resonant plasmonic and metamaterial nanostructures have attracted much attention in the past decade due to their exotic dynamic properties that are not available in nature, such as optical negative refraction^1^,^2^, perfect lensing^3^,^4^ and electromagnetic cloaking^5^. Collective oscillations of free electrons in metals, known as localized or delocalized surface plasmons^6^ lie at the origin of these unique properties, allowing a multitude of exciting applications such as biosensors^7^,^8^,^9^, optical filters^10^, photodetectors^11^ and nanolasers^12^. While the intrinsic optical loss of metals is a major limitation in the performance of these devices, it is advantageous for enhancing light absorption. In 2008, Landy *et al.*^13^ first proposed a perfect metamaterial absorber with nearly perfect absorbance by simultaneously exciting electric and magnetic resonances to realize the impedance match with the surrounding air. After that, substantial absorbers based on different physical mechanisms have been demonstrated theoretically and experimentally in a wide spectral range, which can be generally categorized into broadband absorbers^14^,^15^,^16^ and narrowband absorbers^17^,^18^,^19^,^20^,^21^ in terms of their absorption bandwidth. While broadband absorbers are generally used in thermo-photovoltaics^22^, narrowband perfect absorbers can be used in sensing^9^,^19^,^20^, absorption filtering^23^ and thermal radiation tailoring^24^,^25^.

For sensing applications, Liu *et al.*^9^ experimentally realized a refractive index sensor by using an infrared perfect absorber, indicating that both narrow bandwidth and large modulation depth are necessary to improve the sensing performance. The triple-layer metal-dielectric-metal (MDM) configuration they used has been widely applied to the previous plasmonic absorbers, where a thin dielectric spacer is used to enable strong plasmonic coupling between the top resonators and the bottom metal film. Such an absorber design can also be intuitively treated as a resonator coupled to a single input transmission line, with the dielectric spacer thickness influencing the radiative damping rates and resonant frequency^26^. However, due to the strongly radiative damping and the inherent metal loss, the resonant absorption bandwidths of these plasmonic absorbers are relatively broad (>40 nm), which severely hampers its applications. Thus, it is of great significance to design ultra-narrow band perfect plasmonic absorbers. Up to date, several theoretical and experimental efforts have been devoted to achieve this. Among them, Li *et al.*^18^ experimentally realized a narrow band absorber with an absorption bandwidth of 12 nm and absorptivity exceeding 90% based on surface lattice resonance. Ultra-narrow band perfect absorbers based on a plasmonic analog of electromagnetically induced absorption and optimized grating were theoretically proposed^19^,^20^. In^21^, the authors designed a nanoslit-microcavity-based narrow band absorber with bandwidth of 8 nm and sensing figure of merit (FOM) of 25, which is much higher than the previous sensor with FOM less than 10^17^,^27^,^28^.

In a different context, metal nanostructures based on localized surface plasmonic resonance have generally been effective in creating strongly enhanced electromagnetic fields. Termed electric hot spots, the confined electric field in metal particle junctions enables large enhancement of emission processes and nonlinearities^6^, which are mainly mediated by the electric polarization of molecules. In particular, magnetic activity at optical frequencies is far smaller than its electric counterpart because of the extremely weak magnetic response of natural materials. As a result, magnetic hot spots are highly desirable for strengthening the magnetic response. Many structures have been specifically designed to achieve magnetic hot spots, such as diabolo antennas^29^, two parallel metal plates^30^ and closely spaced thick gold rings^31^. However, achieving simultaneous electric and magnetic hot spots at the same spatial position is rather challenging. Moreover, to maximize the field enhancement, coupled mode theory has recently been used^32^,^33^. Both the quality factor and mode volume are critical parameters in engineering the local field enhancement, while it seems separate between the far-field perfect absorption and the maximum near-field enhancement.

In this paper, we propose an all-metal absorber with an absorption bandwidth less than 8 nm and polarization insensitive absorptivity exceeding 99%. Full-wave electromagnetic simulations reveal that the narrowband perfect absorber with quality factor of 120 can simultaneously create giant electric and magnetic field enhancements in the deep subwavelength scale (mode volumes  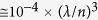). We further demonstrate that perfect absorption is necessary to maximize the local field enhancement based on the coupled mode theory. Additionally, operated as a refractive index sensor, the proposed absorber has a high sensitivity of 885 nm/RIU and figure of merit (FOM) up to 110 in the near infrared region. Our findings provide a new method to engineer narrowband perfect absorption and local field enhancement. It is expected that such absorber structures will hold great potential in sensing and near field optics.

## Results

### Structure and parameters

**Figure 1:**
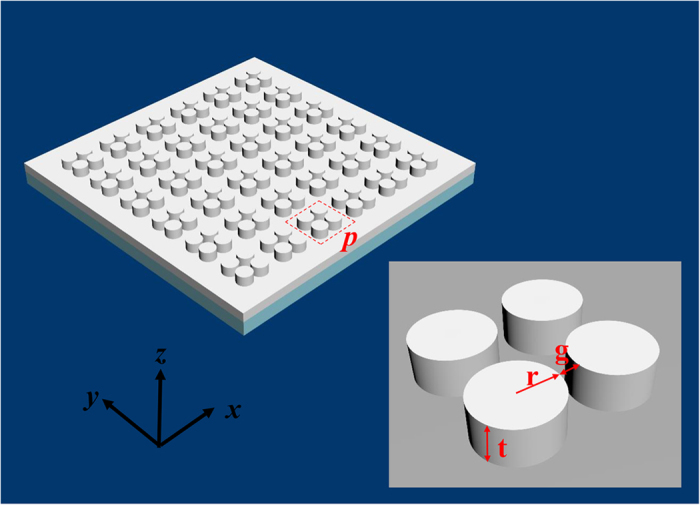
A schematic diagram of the all-metal perfect absorber with a magnified unit cell (enclosed in dashed box) shown in the inset: periodic arrays of coupled thick silver disks are placed directly on the surface of a uniform silver film. The closely spaced silver disks have a radius (*r*) of 80 nm, thickness (*t*) of 100 nm and gap distance (*g*) of 20 nm. The period constant (*p*) of the arrays is 470 nm and the bottom silver film has a thickness of 100 nm. In addition, the whole structure is placed on a glass substrate, and the surrounding material is assumed to be air.

The schematic of the proposed structure is depicted in Fig. 1 with a magnified unit cell (enclosed in dashed box) shown in the inset. The all-metal structure consists of periodic arrays of coupled thick silver disks placed directly on the surface of a uniform silver film. The closely spaced silver disks have a radius (*r*) of 80 nm, thickness (*t*) of 100 nm and gap distance (*g*) of 20 nm. The period constant (*p*) of the arrays is 470 nm and the bottom silver film has a thickness of 100 nm. In addition, the whole structure is placed on a glass substrate, and the surrounding material is assumed to be air. The complex dielectric constant of silver is modeled by a Drude-Lorentz fitting (with 5 coefficients) to tabulate experimental data^34^, and the permittivity of the glass is 2.25. These structures are compatible with the current fabrication technology such as electron beam lithography and focused ion milling.

### Ultra-narrow band perfect absorption based on vertical gap plasmonic mode

**Figure 2:**
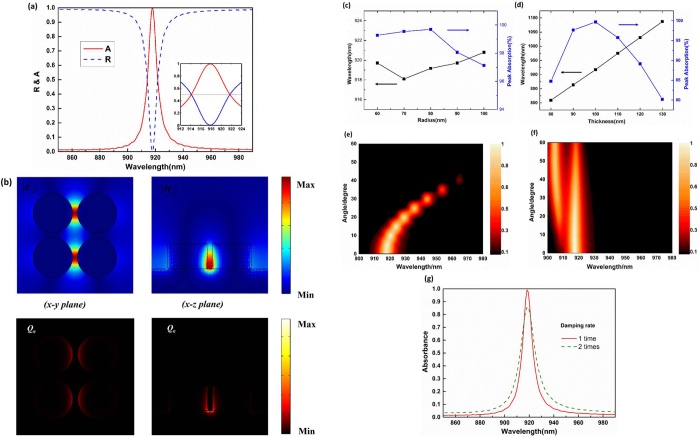
(**a**) The absorption and reflection spectrum with magnified spectrum shown in the inset. (**b**) Distributions of the electric field |***E***_***x***_| (color bar in the x-y plane), magnetic field |***H***_***y***_| (color bar in the x-z plane) and current density (small arrow lines in the x-z plane) at resonance (top row), and mapping of the absorbed power density (bottom row) in both the x-y and x-z planes. Peak absorption and resonant wavelength as the radius (**c**) and thickness (**d**) of the structures varies. Absorbance as a function of the incident angle and wavelength for (**e**) TM-polarized and (**d**) TE-polarized light. (**g**) Simulated absorbance spectra when the damping rate of the silver film is two times that of bulk silver due to the surface scattering and grain boundary effects in a thin film.

Figure 2(a) depicts the absorption and reflection spectrum of the all-metal structures. The spectral absorption (A) is defined by 1-R-T, where R and T are the reflection and transmission of the structures, respectively. Since the thickness of the bottom silver film is thicker than the skin depth in the infrared region, the transmission channel is prevented and the absorption is reduced to 1-R. As shown in Fig. 2(a), there is a distinctive resonance at the wavelength of 918 nm, with the full absorption width at half-maximum of about 7.5 nm (i.e., 0.8% of the central wavelength) and absorptivity of 99.6%. This bandwidth is much narrower than those of MDM based perfect absorbers, whose bandwidths are larger than 40 nm (4% of the central wavelength)^9^,^13^,^17^. Due to the symmetry of the structures, polarization-insensitive absorption can be easily achieved.

To reveal the physical mechanism in the proposed perfect absorber, we calculate the electric, magnetic field (|***E***_***x***_| and |***H***_***y***_|) and current density distributions at the resonant wavelength of 918 nm, and map the absorbed power density in both the x-y and x-z planes of the structure in Fig. 2(b). It is evident that both the electric and magnetic field are strongly concentrated in the gap region as well as the absorbed power density. The resonance oscillates like a magnetic dipole, which can be seen from the confined magnetic field in the gap and the arrow line distribution of the current density in the x-z plane. A vertical Fabry-Perot cavity of MDM waveguide is formed by the disks, and the air gap acts as the dielectric layer^35^,^36^. With the help of the silver film, this resonance can be well excited. Therefore, we can attribute the narrow band perfect absorption to the vertical gap plasmonic mode, due to the relatively poor scattering ability of the magnetic dipole resonance in the deep subwavelength region. To better understand the properties of the vertical gap plasmonic mode, the influences of the radius and thickness of the disks on the absorption spectrum are investigated. The corresponding results are shown in Fig. 2(c,d). As the radius of the disks decreases from 100 nm to 60 nm, the resonant wavelength is slightly shifted, which is consistent with previous studies^35^. By contrast, the resonant wavelength is sensitive to the thickness of the disks, which redshifts from 809 nm to 1087 nm as the thickness is increased from 80 nm to 130 nm. This can be intuitively explained by an increase in the vertical cavity length.

Absorption at oblique incidence for TM and TE polarized waves were also studied and shown in Fig. 2(e,f), respectively. From Fig. 2(e) one sees an obvious red-shift and the decreasing of the absorption peak for TM polarized wave as the incident angle increases. In Fig. 2(f) for TE polarized wave, a second absorption peak emerges and a slight red-shift of the first absorption peak is observed as the incident angle increases. Thus our narrowband perfect absorber can work well only within a narrow incident angle, and this can be regarded as a directional thermal emitter according to Kirchhoff’s law^19^. In addition, due to the surface scattering and grain boundary effects in thin films, the damping constant of the silver film in a real system is likely higher than that of bulk silver. To take this effect into consideration, we simulate the absorption spectra for damping constant of two times that of bulk silver shown in Fig. 2(g). A decreased and broadened absorbance is observed which illustrates that the further increased material loss would degrade the performance of the designed narrowband absorber (the absorption peak may be increased by optimizing slightly the structural parameters with a realistic loss). Recently, an effective method for depositing ultra-smooth and low loss silver films has been demonstrated using a very thin germanium layer (as a wetting material) and a rapid post-annealing treatment^37^,^38^. Thus our narrowband perfect absorber may be fabricated by first depositing a low loss silver film with such an effective method and then patterning with electron beam lithography or focused ion milling.

### Maximum electromagnetic field enhancement based on coupled mode theory

Due to the localized surface plasmonic resonance, plasmonic nanostructures can efficiently link far field radiation with the localized near field, and can be viewed as optical nanoantennas^39^. They are specifically designed to produce large electromagnetic field enhancement within a small mode volume, called a hot spot. In particular, magnetic hot spots are highly desirable for enhancing magnetic dipole transitions and developing magnetic-based devices due to the extremely weak magnetic response of the natural material in the optical domain^31^, while electric hot spots play a crucial role in Surface Enhanced Raman Scattering (SERS), nanolasers and nonlinear optics. Before numerically examining the field enhancement, we first refer to the temporal coupled mode theory which has been used previously to model the field enhancement of an optical antenna^32^,^33^. Consider the structures as optical antennas with an effective radiation cross section of *A*_*c*_, illuminated by an incident beam of cross section *A*_*i*_. The maximum field enhancement at resonance can be expressed^33^ (Supplementary Section S1) as


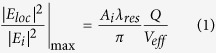


where *E*_*loc*_ and *E*_*i*_ are the local electric field in the gap region and the incident electric field, respectively. *Q* is the quality factor, and *V*_*eff*_ is the effective mode volume of the resonator. The maximum field enhancement is proportional to the Purcell factor^40^ (*Q*/*V*_*eff*_), which can be achieved when simultaneously the radiative decay rate *γ*_*r*_ equals the absorption decay *γ*_*a*_ and the antenna’s radiation pattern matches the incident beam shape, denoted by *γ*_*r*_ = *γ*_*a*_, and *A*_*c*_ = *A*_*i*_. In addition, the critical coupling condition leads to perfect absorption^26^ (see Supplementary Section S2).

**Figure 3:**
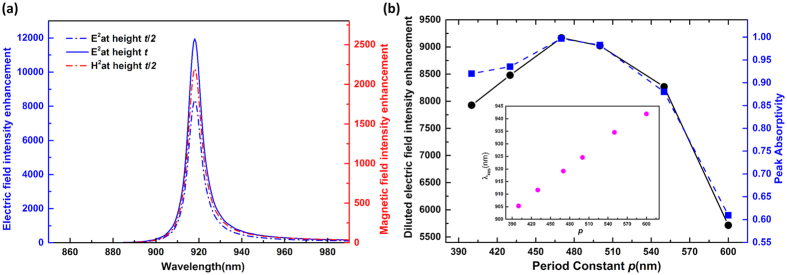
(**a**) Electric (blue dash-dot curve) and magnetic field (red dash-dot curve) intensity enhancement at a point in the middle of the gap and height t/2 over the silver film, and electric field intensity enhancement at height t (blue curve). (**b**) Diluted electric field intensity enhancement (circles connected with solid lines) and the peak absorptivity (squares connected with dashed lines) as the period varies. Inset: resonant wavelength vs period with other parameters unchanged.

The electric and magnetic field intensity enhancement measured at a point in the middle of the gap and at a height *t*/2 over the silver film are shown in Fig. 3(a), and the electric field intensity enhancement at the top surface (height *t*) is also plotted. Both the narrowband electric and magnetic fields are strongly enhanced in the same spatial region (both E and B enhancements are relatively large at a height *t*/2 over the silver film, and their hot spots have spatial overlap at around t/2). This can be ascribed to the vertical gap plasmonic mode. From Fig. 2(b), one sees that the currents (the arrow line distribution in the x-z plane) flow vertically between the thick silver disks through the bottom connected silver film, creating a current loop which gives rise to one-order higher magnetic field than that which would result from putting on the dielectric substrate^31^. This is much like the vertical Split Ring Resonators^41^. Meanwhile, the narrow bandwidth confirms the mode’s high quality factor *Q* to be as high as 120, which is much higher than 20 for the MDM based structures^9^,^33^ and 79 for recently reported ultra-narrow absorbers based on surface lattice resonances^18^. It simultaneously supports a mode volume *V* of 10^−4^ × (*λ*_*res*_/*n*)^3^ at the resonant wavelength of 918 nm in the surrounding material (*n* = 1), which is much smaller than the traditional MDM structures. The high quality factor *Q* and extremely small *V* lead to the observed large electric field enhancement based on the above coupled mode theory.

To validate the relation between field intensity enhancement and absorption, we only change the period constant *p* and keep other parameters the same. This will intuitively influence the ratio of the hot-spot area to the unit cell size, and thus we dilute the field intensity enhancement with the ratio of the hot-spot area to unit cell size as


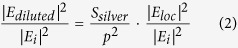


where *S*_*silver*_ is the total top-surface area of the four silver disks in a unit cell. Such a diluted field intensity enhancement is useful to evaluate the performance of multi-hot spot devices^42^,^43^. Figure 3(b) shows the diluted field intensity enhancement and absorption plots as the period *p* varies. We find that the diluted field intensity enhancement is approximately proportional to the absorption, which is consistent with the coupled mode theory, except when the period constant is so small that the near field coupling becomes noticeable and much energy is dissipated between unit cells. Furthermore, the resonant wavelength is slightly shifted as seen from the inset of Fig. 3(b). Therefore, to maximize the local field enhancement, the absorption is more easily engineered compared with the *Q*-matching condition^33^, and perfect absorption is critical.

### Plasmon sensing capability of the structure

**Figure 4:**
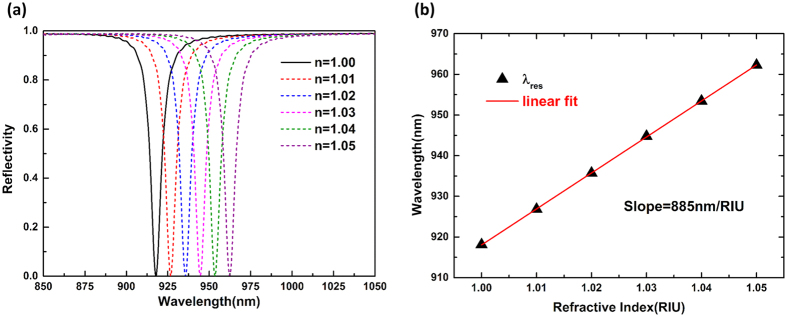
(**a**) Reflection spectrum of the all-metal perfect absorber with the refractive index varying from 1 to 1.05 with a step interval of 0.01. (**b**) Resonant wavelength as a function of the surrounding low refractive index. The red line is the linear fitting with the slope representing the sensitivity S.

As is well known, the resonant wavelength of plasmonic nanostructures is dependent on the refractive index of the surrounding dielectric environment^27^, a property that has been widely utilized for sensing applications. The sensing capability is usually described by the following definitions of sensitivity and figure of merit (FOM)^17^,^21^.


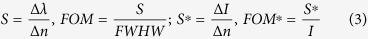


where Δ*λ* is the spectral shift caused by a certain refractive index change in the environment Δ*n*, Δ*I* is the detected intensity change for a particular incident wavelength, and *I* is the absolute intensity. Since our all-metal structure has a narrow bandwidth and nearly zero reflectance (R = 0.44%) around the resonant frequency, it is expected to have good sensing capability. To demonstrate its performance, we vary the surrounding refractive index from 1 to 1.05 with a step interval of 0.01, and the corresponding reflection spectra are displayed in Fig. 4(a), where obvious redshift of the resonance is observed. The slope of the curve in Fig. 4(b) represents the sensitivity *S* of 885 nm/RIU, and an FOM of 110 can be achieved considering its narrow bandwidth. This is much higher than other recently reported values^21^,^27^,^28^,^41^. Furthermore, as seen from Fig. 4(a), a slight spectral shift will cause a large optical intensity variation. We can obtain S^*^ = 85/RIU and FOM^*^ = 19000 at a fixed measurement wavelength of 918 nm from the definition because of the near-unity absorption, and the S^*^ is about one order larger than that of the cavity enhanced localized plasmonic resonance sensing^17^.

## Discussions

In summary, we have proposed an all-metal absorber with an absorption bandwidth less than 8 nm and polarization insensitive absorptivity exceeding 99%. The absorber is based on the localized vertical gap plasmonic mode, unlike traditional MDM configurations. Due to the high quality factor and extremely small mode volume, the perfect absorber can achieve both electric and magnetic hot spots at the same position based on coupled mode theory. We have investigated the relation between the far-field absorption and the localized near-field enhancement, and found that the perfect absorption is critical to maximizing the field enhancement besides the previous *Q*-matching condition. Considering the superior localized plasmonic characteristic of the mode, we demonstrated its sensing capability, and a high sensitivity of 885 nm with FOM up to 110 has been realized. This is much better than most reported values. Our structures can be well tuned over the infrared domain by changing the structure parameters. These findings provide a new design strategy not only for narrow band perfect absorbers but also near field engineering, both electric and magnetic. Such narrowband resonators will easily find applications in thermo-photovoltaics, biosensors, nonlinear plasmonics and lasers.

## Methods

### Simulation

Three-dimensional finite-difference time-domain calculations were performed using a commercially available software package (Lumerical FDTD Solutions Inc.v8.6). Due to the symmetry of the structures, only the plane wave polarized along the x-axis is considered as the excitation source and is incident from the top. Periodic boundary conditions are employed for the lateral boundaries, and perfectly matching layers are applied along the z direction to eliminate the boundary scattering. The mesh size is set to be 0.5 nm which is much smaller than the element sizes and the operating wavelength, and a standard convergence test is done to ensure negligible numerical errors. 2D frequency-domain field and power monitors (perpendicular to the x-y plane) are used to calculate the reflection and transmission, and point monitors are used to record the electromagnetic field enhancement.

### Quality factor and Mode volume

The quality factor was estimated by *Q* = *λ*_*res*_/Δ*λ*, where *λ*_*res*_ is the resonance wavelength, and Δ*λ* is spectral width. The mode volume of the proposed structures was calculated using the formula


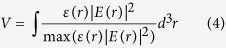


where *ε*(*r*) is the complex dielectric constant at position *r*, and |*E*(*r*)|^2^ is the corresponding electric field intensity^32^. We first calculate the field distribution in the whole structure by using FDTD and then the mode volume V using Eq. (4).

## Additional Information

**How to cite this article**: Yong, Z. *et al.* Narrow band perfect absorber for maximum localized magnetic and electric field enhancement and sensing applications. *Sci. Rep.* **6**, 24063; doi: 10.1038/srep24063 (2016).

## Supplementary Material

Supplementary Information

## References

[b1] Shelby R. A., Smith D. R. & Schultz S. Experimental verification of a negative index of refraction. Science 292, 77–79 (2001). 11292865 10.1126/science.1058847

[b2] Shalaev V. M. Optical negative-index metamaterials. Nature Photon. 1, 41–48 (2007). 10.1038/nature09278 20686570

[b3] Pendry J. B. Negative refraction makes a perfect lens. Phys. Rev. Lett. 85, 3966 (2000). 11041972 10.1103/PhysRevLett.85.3966

[b4] Fang N., Lee H., Sun C. & Zhang X. Sub-diffraction-limited optical imaging with a silver superlens. Science 308, 534 (2005). 15845849 10.1126/science.1108759

[b5] Schurig D. *et al.* Metamaterial electromagnetic cloak at microwave frequencies. Science 314, 977–980 (2006). 17053110 10.1126/science.1133628

[b6] Maier S. A. Plasmonics: Fundamentals and Applications (Springer, 2007).

[b7] Anker J. N. *et al.* Biosensing with plasmonic nanosensors. Nature Mater. 7, 442–453 (2008). 18497851 10.1038/nmat2162

[b8] Kabashin A. V. *et al.* Plasmonic nanorod metamaterials for biosensing. Nature Mater. 8, 867–871 (2009). 19820701 10.1038/nmat2546

[b9] Liu N., Mesch M., Weiss T., Hentschel M. & Giessen H. Infrared perfect absorber and its application as plasmonic sensor. Nano Lett. 10, 2342–2348 (2010). 20560590 10.1021/nl9041033

[b10] Ellenbogen T., Seo K. & Crozier K. B. Chromatic plasmonic polarizers for active visible color filtering and polarimetry. Nano Lett. 12, 1026–1031 (2012). 22229785 10.1021/nl204257g

[b11] Sobhani A. *et al.* Narrowband photodetection in the near-infrared with a plasmon-induced hot electron device. Nature Commun. 4, 1643 (2013). 23535664 10.1038/ncomms2642

[b12] Oulton R. F. *et al.* Plasmon lasers at deep subwavelength scale. Nature 461, 629–632 (2009). 19718019 10.1038/nature08364

[b13] Landy N. I., Sajuyigbe S., Mock J. J., Smith D. R. & Padilla W. J. Perfect metamaterial absorber. Phys. Rev. Lett. 100, 207402 (2008). 18518577 10.1103/PhysRevLett.100.207402

[b14] Aydin K., Ferry V. E., Briggs R. M. & Atwater H. A. Broadband polarization-independent resonant light absorption using ultrathin plasmonic super absorbers. Nature Commun. 2, 517 (2011). 22044996 10.1038/ncomms1528

[b15] Ding F., Cui Y., Ge X., Jin Y. & He S. Ultra-broadband microwave metamaterial absorber. Appl. Phys. Lett. 100, 103506 (2012).

[b16] Cui Y. *et al.* Ultrabroadband light absorption by a sawtooth anisotropic metamaterial slab. Nano Lett. 12, 1443–1447 (2012). 22309161 10.1021/nl204118h

[b17] Ameling R. *et al.* Cavity-enhanced localized plasmon resonance sensing. Appl. Phys. Lett. 97, 253116 (2010).

[b18] Li Z., Butun S. & Aydin K. Ultranarrow band absorbers based on surface lattice resonances in nanostructured metal surfaces. ACS Nano 8, 8242–8248 (2014). 25072803 10.1021/nn502617t

[b19] Meng L. *et al.* Optimized grating as an ultra-narrow band absorber or plasmonic sensor. Opt. Lett. 39, 1137–1140 (2014). 24690690 10.1364/OL.39.001137

[b20] He J., Ding P., Wang J., Fan C. & Liang E. Ultra-narrow band perfect absorbers based on plasmonic analog of electromagnetically induced absorption. Opt. Express 23, 6083–6091 (2015). 25836832 10.1364/OE.23.006083

[b21] Lu X., Zhang L. & Zhang T. Nanoslit-microcavity-based narrow band absorber for sensing applications. Opt. Express 23, 20715–20720 (2015). 26367923 10.1364/OE.23.020715

[b22] Atwater H. A. & Polman A. Plasmonics for improved photovoltaic devices. Nature Mater. 9, 205–213 (2010). 20168344 10.1038/nmat2629

[b23] Lee K. T., Seo S. & Guo L. J. High-color-purity subtractive color filters with a wide viewing angle based on plasmonic perfect absorbers. Adv. Opt. Mater. 3, 347–352 (2015).

[b24] Liu X. *et al.* Taming the blackbody with infrared metamaterials as selective thermal emitters. Phys. Rev. Lett. 107, 045901 (2011). 21867022 10.1103/PhysRevLett.107.045901

[b25] Song M. *et al.* Conversion of broadband energy to narrowband emission through double-sided metamaterials. Opt. Express 21, 32207–32216 (2013). 24514815 10.1364/OE.21.032207

[b26] Wu C. *et al.* Large-area wide-angle spectrally selective plasmonic absorber. Phys. Rev. B 84, 075102 (2011).

[b27] Becker J., Trügler A., Jakab A., Hohenester U. & Sönnichsen C. The optimal aspect ratio of gold nanorods for plasmonic bio-sensing. Plasmonics 5, 161–167 (2010).

[b28] Huang C., Ye J., Wang S., Stakenborg T. & Lagae L. Gold nanoring as a sensitive plasmonic biosensor for on-chip DNA detection. Appl. Phys. Lett. 100, 173114 (2012).

[b29] Grosjean T., Mivelle M., Baida F. I., Burr G. W. & Fischer U. C. Diabolo nanoantenna for enhancing and confining the magnetic optical field. Nano Lett. 11, 1009–1013 (2011). 21319837 10.1021/nl103817f

[b30] Cai W. S. *et al.* Metamagnetics with rainbow colors. Opt. Express 15, 3333–3341 (2007). 19532574 10.1364/oe.15.003333

[b31] Lorente-Crespo M. *et al.* Magnetic hot spots in closely spaced thick gold nanorings. Nano Lett. 13, 2654–2661 (2013). 23688257 10.1021/nl400798s

[b32] Maier S. A. Plasmonic field enhancement and SERS in the effective mode volume picture. Opt. Express 14, 1957–1964 (2006). 19503526 10.1364/oe.14.001957

[b33] Seok T. J. *et al.* Radiation engineering of optical antennas for maximum field enhancement. Nano Lett. 11, 2606–2610 (2011). 21648393 10.1021/nl2010862

[b34] Johnson P. B. & Christy R. W. Optical constants of the noble metals. Phys. Rev. B 6, 4370 (1972).

[b35] Bozhevolnyi S. I. & Søndergaard T. General properties of slow-plasmon resonant nanostructures: nano-antennas and resonators. Opt. Express 15, 10869–10877 (2007). 19547444 10.1364/oe.15.010869

[b36] Le Perchec J., Quemerais P., Barbara A. & Lopez-Rios T. Why metallic surfaces with grooves a few nanometers deep and wide may strongly absorb visible light. Phys. Rev. Lett. 100, 066408 (2008). 18352499 10.1103/PhysRevLett.100.066408

[b37] Logeeswaran V. J. *et al.* Ultrasmooth silver thin films deposited with a germanium nucleation layer. Nano Lett. 9, 178–182 (2008). 10.1021/nl8027476 19105737

[b38] Chen W., Thoreson M. D., Ishii S., Kildishev A. V. & Shalaev V. M. Ultra-thin ultra-smooth and low-loss silver films on a germanium wetting layer. Opt. Express 18, 5124–5134 (2010). 20389525 10.1364/OE.18.005124

[b39] Novotny L. & Van Hulst N. Antennas for light. Nature Photon. 5, 83–90 (2011).

[b40] Purcell E. M. Spontaneous transition probabilities in radio-frequency spectroscopy. Phys. Rev. 69, 681 (1946).

[b41] Wu P. C. *et al.* Vertical split-ring resonator based nanoplasmonic sensor. Appl. Phys. Lett. 105, 033105 (2014).

[b42] Chu Y., Banaee M. G. & Crozier K. B. Double-resonance plasmon substrates for surface-enhanced Raman scattering with enhancement at excitation and stokes frequencies. ACS Nano 4, 2804–2810 (2010). 20429521 10.1021/nn901826q

[b43] Linden S. *et al.* Collective effects in second-harmonic generation from split-ring-resonator arrays. Phys. Rev. Lett. 109, 015502 (2012). 23031113 10.1103/PhysRevLett.109.015502

