# Supplementary material for: Narrow band perfect absorber for maximum localized magnetic and electric field enhancement and sensing applications
Source: Sci Rep. 2016 Apr 5;6:24063. doi: 10.1038/srep24063 (PMC4820729; doi:10.1038/srep24063)
Supplement: Supplementary Information [file srep24063-s1.pdf]

Supplementary Information

Narrow band perfect absorber for maximum localized magnetic and electric field enhancement and sensing applications

Zhengdong Yong[1], Senlin Zhang[1], Chengsheng Gong[1], and Sailing He[*, 1, 2]

[1]Centre for Optical and Electromagnetic Research, State Key Laboratory of Modern Optical Instrumentations, Zhejiang University, Hangzhou 310058, China.

[2]Department of Electromagnetic Engineering, School of Electrical Engineering, Royal Institute of Technology (KTH), S-100 44 Stockholm, Sweden

*** Email for the corresponding author: sailing@kth.se**

Section S1：Derivation of the maximum local field enhancement based on couple mode theory

Consider a resonator illuminated by an incident beam of cross section A_i , the resonance amplitude a is governed by the temporal coupled mode theory[1] as:

$$\frac{d}{dt}a = j\omega_0 a - \frac{\gamma_a}{2}a - \frac{\gamma_r}{2}a + kS_+ \qquad \text{(S1)}$$

where $|S_+|^2 = \frac{1}{2\eta}A_i|E_i|^2$ is the power of the excitation beam, ω_0 is the resonant frequency of the resonator and γ_a , γ_r are the energy decay rates due to absorption and radiation, respectively, and k is the coupling coefficient between the resonator and external illumination, which can be expressed as $k = \sqrt{(A_c/A_i)\gamma_r}$. Here A_c is the effective radiation cross section and assumed to be smaller than A_i . With the time-harmonic field of frequency ω , the above equation gives

$$|a|^2 = \frac{(A_c/A_i)\gamma_r|S_+|^2}{(\omega-\omega_0)^2 + (\gamma_a/2+\gamma_r/2)^2} \qquad \text{(S2)}$$

The resonance amplitude a is related to the local field amplitude $|E_{loc}|$ in the resonator by $|a|^2 = \frac{1}{2}\varepsilon_0 |E_{loc}|^2 V_{eff}$, where V_{eff} is the effective mode volume. Thus we can get the field enhancement at resonance as[2]:

$$\frac{|E_{loc}|^2}{|E_i|^2} = \frac{4A_c c\gamma_r}{\omega_0^2}\frac{Q^2}{V_{eff}} \tag{S3}$$

To obtain the maximum field enhancement, the effective radiation cross section A_c needs to be optimized through spatial mode matching ($A_c = A_i$). Furthermore, since the γ_a and V_{eff} are varying slowly with respect to γ_r around resonant frequency ω_0, by simply taking $\partial(|E_{loc}|^2/|E_i|^2)/\partial\gamma_r = 0$, one can see that $\frac{|E_{loc}|^2}{|E_i|^2}$ is maximum when $\gamma_r = \gamma_a$ [3]. Thus the maximum field enhancement can be expressed as

$$\left.\frac{|E_{loc}|^2}{|E_i|^2}\right|_{\max} = \frac{A_i\lambda_{res}}{\pi}\frac{Q}{V_{eff}} \tag{S4}$$

which is shown in the main text.

Section S2: Derivation of the relation between absorption and local field enhancement

The part of the excitation plane wave which can be directly coupled to the antenna mode is denoted by $S_{+coupled}$ (the amplitude of the mode), and the other part of excitation is denoted by $S_{+uncoupled}$, with the relations $|S_+|^2 = |S_{+coupled}|^2 + |S_{+uncoupled}|^2$ and $|S_{+coupled}|^2/|S_+|^2 = A_c/A_i$. The reflected power $|S_{ref}|^2$ can be expressed as $|S_{ref}|^2 = |S_{-uncoupled}|^2 + |S_{-coupled}|^2$, where $S_{-uncoupled} = S_{+uncoupled}$, and $S_{-coupled} = -S_{+coupled} + \gamma_r a$ according to the temporal coupled mode theory. Then we can get the absorption A =1-R at resonance, which can be expressed as[1, 3]:

$$A = 4\frac{A_c}{A_i}\frac{\gamma_r}{\gamma_r+\gamma_a}\frac{\gamma_a}{\gamma_r+\gamma_a} = \frac{\gamma_a \cdot V_{eff}}{c \cdot A_i} \cdot \frac{|E_{loc}|^2}{|E_i|^2} \tag{S5}$$

The perfect absorption condition is achieved when $A_c / A_i = 1$ and $\gamma_r = \gamma_a$ simultaneously, same as that of maximum field enhancement. Furthermore, if γ_a and V_{eff} vary slowly, the absorption is approximately proportional to the diluted field enhancement $\frac{S_{silver}}{A_i} \cdot \frac{|E_{loc}|^2}{|E_i|^2}$, which is defined in the main text.

References:

1. Joannopoulos, J. D., Johnson, S. G., Winn, J. N., & Meade, R. D. *Photonic crystals: molding the flow of light* (Princeton Univ. Press, 2011).

2. Maier, S. A. Plasmonic field enhancement and SERS in the effective mode volume picture. *Opt. Express* ***14***, 1957-1964 (2006).

3. Seok, T. J. *et al*. Radiation engineering of optical antennas for maximum field enhancement. *Nano Lett*. ***11***, 2606-2610 (2011).
